# Barriers to and facilitators of endorsement for scheduled medicines in podiatry: a qualitative descriptive study

**DOI:** 10.1186/s13047-021-00457-9

**Published:** 2021-03-10

**Authors:** Kristin Graham, Helen A. Banwell, Ryan S. Causby, Saravana Kumar, Esther Jie Tian, Lisa Nissen

**Affiliations:** 1 Allied Health & Human Performance, The University of South Australia, North Terrace, Adelaide, SA 5000 Australia; 2 Faculty of Health, School, Clinical Sciences, Queensland University of Technology, Brisbane, Australia

**Keywords:** Endorsement for scheduled medicines, Qualitative, Barriers, Facilitators, Podiatrists, Podiatry, Scope of practice, Non-medical prescriber

## Abstract

**Background:**

Australian podiatrists and podiatric surgeons who have successfully completed the requirements for endorsement for scheduled medicines, as directed by the Podiatry Board of Australia, are eligible to prescribe a limited amount of schedule 2, 3, 4 or 8 medications. Registration to become endorsed for scheduled medicines has been available to podiatrists for over 10 years, yet the uptake of training has remained low (approximately 2% of registered podiatrists/podiatry surgeons). This study aimed to explore barriers to and facilitators of engagement with endorsement for scheduled medicines by podiatrists.

**Methods:**

Qualitative descriptive methodology informed this research. A purposive maximum variation sampling strategy was used to recruit 13 registered podiatrists and a podiatric surgeon who were either endorsed for scheduled medicines, in training or not endorsed. Semi-structured interviews were employed to collate the data which were analysed using thematic analysis.

**Results:**

Three overarching super-ordinate themes were identified which encompassed both barriers and facilitators: (1) competence and autonomy, (2) social and workplace influences, and (3) extrinsic motivators. Within these, several prominent sub-themes emerged of importance to the participants including workplace and social networks role in modelling behaviours, identifying mentors, and access to supervised training opportunities. Stage of life and career often influenced engagement. Additionally, a lack of financial incentive, cost and time involved in training, and lack of knowledge of training requirements were influential barriers. Rural podiatrists encountered a considerable number of barriers in most of the identified areas.

**Conclusion:**

A multitude of barriers and facilitators exist for podiatrists as part of the endorsement for scheduled medicines. The findings suggest that a lack of engagement with endorsement for scheduled medicines training may be assisted by a more structured training process and increasing the number of podiatrists who are endorsed to increase the numbers of role models, mentors, and supervision opportunities. Recommendations are provided for approaches as means of achieving, and sustaining, these outcomes.

## Background

In 2010, legislative changes enabled the Podiatry Board of Australia to register Australian podiatrists and podiatric surgeons to use a range of drugs to treat podiatric conditions. Podiatrists who completed training in endorsement for scheduled medicines could register to administer, obtain, possess, prescribe, sell, supply or use a limited range of Schedule 2, 3, 4 or 8 medicines for the treatment of podiatric conditions if included in the *National Podiatry Scheduled Medicines List* [1], within the limits of the relevant State and Territory legislation [2]. The opportunity for allied health professionals to extend their scope of practice to include prescribing scheduled medicines was developed in response to the ever-increasing demand on health services. Furthermore, non-medical prescriber status has proven to improve patient satisfaction and outcomes, be cost-effective [3], and safe [4–6].

Podiatrists and podiatric surgeons are involved in the management of several conditions that may benefit from the timely prescription of scheduled medicines, including anxiety surrounding specific interventions, painful inflammatory presentations, and skin and soft tissue infection [7], including those related to diabetes-related foot disease or the high-risk foot. Delays in therapeutic intervention can result in further complications, including the potential for critical colonisation of foot ulcers, and/or osteomyelitis increasing the risk of lower limb amputation [8]. Podiatrists are well placed to identify, intervene, and manage complex and high-risk foot presentations.

**Fig. 1:**
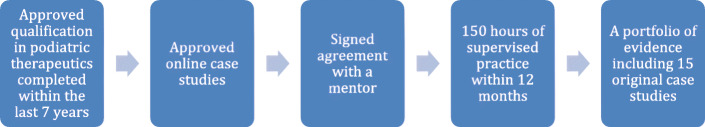
Flow chart of the Pathway B to endorsement for scheduled medicines

There are two pathways for podiatrists and podiatric surgeons to become endorsed prescribers of scheduled medicines (endorsed prescribers). Until recently, Pathway A (Historic Pathway A) addressed the needs of podiatrists who held an approved qualification for endorsement for scheduled medicines [8], for example podiatric surgeons. However, Pathway A has been amended to incorporate approved programs of study for endorsement for scheduled medicines which includes substantial exposure in the prescription of specified medicines. Whilst the legislation for this pathway has been passed, at the time of writing this manuscript there are currently no approved programs of study for the new Pathway A as the accreditation guidelines are still under development [9]. Pathway B is via supervised practice which involves completion of a Podiatry Board of Australia approved university qualification in podiatric therapeutics and submission of a portfolio measured against a national competency framework (see Fig. 1) [9]. The portfolio includes 150 hours of supervised practice within a variety of health settings and a mentored case study log of 15 cases describing exposure across the classes of medications included on the scheduled medicines list for podiatry [9].

Many universities now include an approved qualification in podiatric therapeutics as part of undergraduate education. For those who have not complete an undergraduate course, at the time of publication two Australian universities offered approved programs in podiatric therapeutics designed specifically for Pathway B. Both courses are part-time and take between 6 and 12 months to complete at approximate costs of between $6200 (University of South Australia, Advanced Pharmacology for Podiatrists [10]) and $11,600 (Queensland University of Technology, Graduate Certificate in Podiatric Therapeutics [11]).

This endorsement process aligns podiatrists with other non-medical prescribers such as optometrists, pharmacists, and nurse practitioners [12]. The uptake of endorsed prescriber status with the podiatry profession is approximately 2% (127 of 5584) [13], considerably less than that of optometrists (64.9%) [14], but similar to nurse practitioners at 1% (including midwives and nurses with rural and remote scheduled medicine endorsement) [15].

Given the benefits of podiatrists becoming endorsed prescribers, it raises the question of why the uptake remains so low within the profession? Time and supervision requirements have been purported to impact on uptake [3]; however, to the best of the Authors’ knowledge, despite extensive anecdotal discussion, little investigation has been conducted into the factors that influence podiatrists’ engagement with endorsed podiatry prescriber status. Therefore, the primary aim of this study was to explore barriers and facilitators to podiatrists engaging with the endorsement for scheduled medicines. No a priori hypotheses were made about the themes that would be uncovered.

## Methods

### Methodology

A qualitative descriptive (QD) methodology [16] was chosen to explore and gain an understanding of various barriers and facilitators to endorsement for podiatrists, which could then be used to address important knowledge gaps in this field. This method examines descriptions of individual’s characteristics, traits, and behaviours that occur in everyday context using common language [16, 17] to provide a precise portrayal of the phenomenon of interest. As a result, the findings produced are close to the collected data and within an identifiable local context.

### Study participants and selection procedures

Potential participants included current practicing podiatrists or podiatric surgeons registered with the Australian Health Practitioner Regulation Agency (AHPRA). As means of capturing varied perspectives from podiatrists with diverse characteristics (personal, professional, and clinical), participants from a broad range of practice settings, years of professional experience, diversity of roles, and registration status for endorsement were identified through the research team’s extensive personal and professional networks and invited to participate via email or phone.

Given the exploratory nature of qualitative research, the purpose of sampling is not focused on representation of the population but rather gaining a deep understanding of the phenomena of interest. Sampling and sample size can be influenced by methodological and practical considerations [17, 18]. Methodological considerations include variability within the sample, diversity of issues, and ensuring data collection continues until consistency in the responses are attained. Practical consideration included budgets, time and other resources required to undertake data collection, and analysis [17, 19]. Therefore, this study utilised a purposive maximum variation sampling strategy as means of capturing wide ranging perspectives and to explore the phenomena from different viewpoints. By doing so, this research was able to identify diverse variations and map common patterns that may exist across variations.

### Data collection

All data were collected via individual, semi-structured interviews to obtain in-depth and independent understanding of participant’s perspectives [20]. The interviews were conducted by two members of the research team (ET or SK) who have expertise in allied health practice and research and previous experience in conducting semi-structured interviews but were not known to participants either professionally or personally. Given that podiatry is a relatively small profession in Australia, engaging members of the research team who were not podiatrists but had an allied health background (SK is a physiotherapist and ET is a dietitian) maintained rigour during the data collection process. An interview guide was developed by the members of the research team (KG, HB, RC, and LN) with extensive experience in clinical practice and research and piloted with an external group of podiatrists. The interview guide comprised a wide range of open-ended questions with opportunities for prompts [18]. The researcher sought additional clarifications if further elaboration was needed from participants. Each interview lasted approximately 20–30 min and was conducted via telephone in a secure room. All interviews were audio-recorded (with permission from participants) and transcribed verbatim by an independent third party.

### Data analysis

Thematic analysis was chosen for data analysis in this study [21]. The coding process was discussed within the research team prior to the conduct of data analysis to reach a consensus. Once this had been established, the coding process was undertaken manually by one member of the research team (ET). Each interview transcript was read more than once by the researcher and ideas generated from this process were labelled as codes. The same process was repeated across all transcripts and common codes were identified and categorised to form themes [20, 22]. These themes were subsequently labelled based on messages that they presented. A second member of the research team (SK) was consulted in an ongoing manner when, if any, uncertainties were identified during the coding and theming. The research team identified super-ordinate themes emergent from the data. The data analysis was informed by the self-determination theory (SDT), a broad framework for the study of human motivation often applied in the educational context [23]. SDT posits that an individual’s experience of autonomy and competence are important in fostering motivation and engagement with activities. SDT, however, also recognises that supporting continued engagement requires ongoing social supports and there may be other extrinsic motivators which may also play an influencing role, albeit to a lesser level.

As a means of enhancing the rigour of the qualitative data collection, analysis, and interpretation processes, a range of strategies were employed to promote credibility, transferability, dependability, and confirmability. These included adherence to the semi-structured interview guide, audiotaping interviews, transcribing verbatim by an independent and external typist, and cross checking within the research team [23, 24]. These processes had been used previously [17, 19] and thus were familiar to the research team. All data were de-identified to promote trustworthiness of the data analysis process. Prior to the commencement of the study, an independent review of the research processes was undertaken by the Human Research Ethics Committee.

## Results

Participant characteristics are described in Table 1. Twelve podiatrists and one podiatric surgeon were recruited into the study. Of the 13 participants 9 were female with a median of 13.5 years of podiatry practice (range 1 to > 30) reported. Most participants were based in South Australia, with two from Queensland and one each from Western Australia and Victoria. Participants worked predominantly in private practice, with two identifying public sector workplaces, and one person working in academia. The majority of participants were based in metropolitan practices, 3 identified as rural or regional practitioners. Of the 13 participants, three were endorsed prescribers and three were in training. One participant, P1, completed endorsement under Historic Pathway A [8]. Of the participants who were non-endorsed, three were willing to become endorsed, two were neutral on the subject, one was not willing to become endorsed, and one participant preference was not reported.

**Table 1:** Participant characteristics

Participant ID	Years since graduation	State of practice	Type of employment	Region of employment	Endorsed prescriber status and pathway to endorsement
P1	> 30	VIC	Full-time	Metropolitan and regional	Endorsed (Historic Pathway A)
P2	3	SA	Full-time	Metropolitan	In-training (Pathway B)
P3	8	SA	Part-time	Metropolitan	In-training (Pathway B)
P4	8	QLD	Full-time	Metropolitan	Endorsed (Pathway B)
P5	2	WA	Part-time	Metropolitan	In-training (Pathway B)
P6	10	SA	Full-time	Metropolitan	Non-endorsed
P7	12	SA	Full-time	Rural and regional	Non-endorsed
P8	8	SA	Full-time	Metropolitan	Non-endorsed
P9	> 30	SA	Full-time	Rural and regional	Non-endorsed
P10	28	SA	Full-time	Metropolitan	Non-endorsed
P11	1	SA	Part-time	Metropolitan	Non-endorsed
P12	20	SA	Part-time	Rural and regional	Non-endorsed
P13	16	QLD	Full-time	Metropolitan	Endorsed (Pathway B)

*SA* South Australia, *VIC* Victoria, *QLD* Queensland, *WA* Western Australia

### Super-ordinate themes

Three super-ordinate themes were found that incorporated both the barriers and facilitators and are listed with their sub-themes in Table 2. A description of each super-ordinate theme is provided below with supporting quotes.

**Table 2:** Sub-themes and super-ordinate themes

Super-ordinate themes	Subthemes
Competence and autonomy	Facilitators: Broaden scope of practice & better patient care Rural location Prior knowledge Job security and competitiveness Not wanting to be left behind Barriers: Conservatism- no perceived need for the increased scope of practice Concern it may compromise the level of competence
Social and workplace influences	Facilitators and barriers: Access to mentors Access to supervised training opportunities Workplace setting Workplace and social networks Facilitators: Working with an endorsed prescriber Barriers: Small number of podiatry prescribers Lack of patient awareness and stigma of being ‘toenail cutters’ Lack of formalised structure Easy access to other prescribers Rural location
Extrinsic motivators	Barriers: Time Stage of life and career Lack of Financial reward Lack of PBS funding Knowledge of the process Endorsement not completely incorporated into undergraduate training Facilitators: SARS-CoV-2 (COVID-19)

#### Theme 1: competence and autonomy

Feeling competent as a practitioner and developing a broader scope of practice were highly reported themes. The drive for competence was closely related to a desire for autonomy, or a sense of being able to take direct action that will improve a patient’s outcome. Participants described complete patient care and improved patient outcomes as being central to a desire for autonomy.

*“Yes, I would like to become an endorsed prescriber ….because I'd like to broaden my scope of practice and have the opportunity to provide pharmacological benefits to my patients… and give my patients better outcomes with providing sort of high level of care”* ***P11*** *(non-endorsed; willing to become endorsed; private sector; metro based)*

Competence and autonomy were commonly reported as an important facilitator by those either already endorsed or currently in training, suggesting this is a motivation that empowered action.

*“My main motivation was really because I was doing a lot of ingrown toenail procedures and one of the more common adverse events is infection, postoperative infection… you can only manage it [conservatively] so far.”* ***P13*** *(endorsed pod; experience in public and private sectors; metro based)*

A desire for autonomy was particularly strong in those based in rural environments where participants observed poor access for patients to general practitioners (GPs).

*“…the main reason I guess [to become endorsed] is that because we are rural… We have very few doctors in the last year or in the last 18 months, there's been a shortage in our area……..I feel that if I'm in a place that I can prescribe antibiotics straight away, that would be more beneficial for my patients”* ***P12*** *(non-endorsed; willing to become endorsed; private sector; regional/rural and metro based)*

Individuals who already had a level of competence with pharmacological knowledge recognised the advantage of this prior knowledge and expressed a desire to capitalise on this.

*“… Also my background in pharmacy did help a lot. I was familiar with the majority of drug names, even those that aren't on our list and how pharmacists work, that helped a lot.”* ***P4*** *(endorsed pod; private sector; metro based)*

An approved qualification in podiatric therapeutics is now incorporated into most undergraduate courses. One participant demonstrated that this study could provide a sense of competence that may motivate further skill development.

*“because I'm more fresh (sic) out of university, I'd like to do it sort of sooner rather than later so that my knowledge that I gained through university, won't be lost.”* ***P11*** *(non-endorsed; willing to become endorsed; private sector; metro based)*

Even in a participant unwilling to become endorsed, increased pharmacological knowledge was recognised to provide valuable insight in patient care.

*….. I wasn't that keen on pharmacology at uni, but I could definitely see the advantages of it once I got out in practice...”* ***P9*** *(non-endorsed; unwilling to become endorsed; private sector; regional/rural based)*

Some participants believed that becoming an endorsed prescriber may improve job security.

*“…I thought professionally it would make me a more desirable job applicant…….it increases job security, having that extra skill.”* ***P2*** *(completed training; public sector; metro based)*

Similarly, one participant expressed a desire not to be left behind the skill progression in the profession.

*“…I guess I decided to do it because I can see that that's the direction the podiatric discipline is heading and I don't want to be left behind, so I assume that eventually students will graduate with the ability to prescribe and that would put me at a disadvantage in the future.”* ***P5*** *(early stage training; private sector; metro based)*

On the other hand, some podiatrists demonstrated an innate conservatism in professional identity and boundaries. These podiatrists believed they could be successful without becoming endorsed or they practiced in an area of podiatry that they perceived did not require an increased scope of practice provided by becoming an endorsed prescriber.

*“I don't think people really see the benefit because you can be a successful podiatrist without the need for having endorsed capabilities. I also think that some podiatrists have chosen a different path, so they're steering away from a medical-based podiatrist and heading more towards a sports podiatrist or biomechanics, in which case it's also not necessarily required to have the endorsed prescribing.”* ***P5*** *(early stage training; private sector; metro based)*

*“…When I first graduated from university, I went straight to aged care. So, in the aged care sector, it was all nursing homes and home visits. So there was no need to study for prescribing rights…”* ***P8*** *(non-endorsed; neutral; private sector; metro based)*

Further to this, one participant reflected that having access to scheduled medicines may decrease competence and result in a deterioration of the level of skill in the profession. This participant exposed a deep respect for traditional podiatry skills and suggested they would rather avoid the temptation of access to scheduled medicines.

*“a problem with being a prescriber is much like a lot of general practices…prescribe drugs become the first avenue of action, instead of the last avenue of action. So I have my concerns about that process as well, that we lose our ability to work without them, or it becomes easy to work with them…”* ***P7*** *(non-endorsed; unwilling to become endorsed; private sector; regional/rural based)*

#### Theme 2: social and workplace influences

Analysis of the data revealed both social and workplace culture and beliefs also influenced engagement, behaviours, and values.

The low numbers of podiatrists that hold endorsement were a prominent barrier in many ways including creating the perception that it is not a ‘necessary’ skill required to practice.

*“… I think when more podiatrists do it [prescribing]…At the moment there's not many, but... If it becomes a necessity and everyone needs to get it, then it might entice more people to train for it.”* ***P8*** *(non-endorsed; neutral; private sector; metro based)*

Whereas, when prescribers were visible such as working with someone who was an endorsed prescriber, it exposed podiatrists to the benefits of having this skill.

*“I think having an endorsed prescriber at my workplace full time as my main supervisor was a very big help….. that would have been the primary one [facilitator]”* ***P4*** *(endorsed pod; private sector; metro based)*

Further, having a supportive workplace that provided opportunity and flexibility was a commonly reported facilitator.

*“…I had a very supportive boss that allowed me to go and sit in with all these other disciplines….and gave me flexibility around work time to do those extra activities as well.”* ***P2*** *(completed training; public sector; metro based)*

Mentors play a pivotal role in the process to gaining endorsement and access to one was an important facilitator. However, for many identifying a mentor was a barrier. One participant highlighted that access to mentors is a symptom of the lack of a formalised structure in the current pathway to endorsement.

*“…The next big one is the formulary that we have. And there's not enough opportunities…for clinical training in a closely mentored environment…If you don't have a formalised sort of residency type or intern structure that people can plug in to, they feel kind of nervous about doing it… The difficult bit is that clinical competency and [the] way you can get it developed. That's not very well addressed to this at this stage…”* ***P1*** *(endorsed podiatric surgeon; private sector; metro based)*

Access to opportunities to undertake the supervised practice component of the training was widely reflected upon. Like mentor access, easy access through the workplace, was a facilitator.

*“… because I was working in the public sector, I had access to rotations with other health practitioners really easily. So, I could go and sit in on endocrinologists or rheumatologists or contact pharmacists or vascular surgeons. I had all of these disciplines that I already had strong relationships with, so I could just go and…spend some time with them to develop these cases. I think that was a really key factor for me…”* ***P13*** *(endorsed podiatrist; experience in public and private sectors)*

However, a lack of access to these opportunities was recognised by participants as a barrier for those outside of the hospital system in fulfilling the required supervised practice across broad areas of medicine.

*“…I think the biggest barrier to the process and starting it, is finding someone in the first place. And then also, because I work in XXXXX (a large public sector organisation), I'm covered by professional indemnity insurance through XXXXX (a large public sector organisation) and if you are a private podiatrist and wanted to observe at a hospital, it becomes very difficult because of mandatory training, confidentiality, hospital insurance…..I think that's a huge barrier for other podiatrists.”* ***P2*** *(completed training; public sector; metro based)*

There were also examples of how both social and workplace networks can undermine people’s sense of volition and initiative to become an endorsed prescriber.

*“…with our podiatry peers, you don't really talk about it much. So, I guess it's not something that's big on our agenda at the moment, or has been since I've graduated, I only know one person that's got prescribing rights in my podiatry peers.”* ***P8*** *(non-endorsed; neutral; private sector; metro based)*

Additionally, professional networks can provide easy access to alternate prescribers, negating the need to become endorsed.

*“…I have good relationships with the local general practitioners and so most things that need prescribing can be easily attained through that relationship…”* ***P7*** *(non-endorsed; unwilling to become endorsed; private sector; regional/rural based)*

Participants also reflected that some work environments may be more supportive of the endorsement process. Some in private practice thought those in the public sector had more support to complete endorsement, while others thought the easy access to medical prescribers may discourage public sector podiatrists from becoming endorsed.

*“I think in the public sector at the moment, you have a lot more time and then seem to have a lot more allocation for continued education and development, so now they can allocate their time easier……They've paid time to do these sorts of things…”* ***P7*** *(non-endorsed; unwilling to become endorsed; private sector; regional/rural based)*

*“…I probably would be discouraged in public [sector] in that…you have access to other people who can prescribe so…… So they may not see that as a number one priority…”* ***P9*** *(non-endorsed; unwilling to become endorsed; private sector; regional/rural based)*

Similarly, one private practice podiatrist thought that being an endorsed prescriber would be more useful in private than in public practice.

*In private, I can sort of, I can see that it would be of more benefit …* ***P9*** *(non-endorsed; unwilling to become endorsed; private sector; regional/rural based)*

In addition to limited mentoring and supervised practice opportunities, the small number of endorsed prescribers may result in a lack of awareness on a public level of the scope of practice of modern podiatry. One participant commented on the stigma of podiatrists being ‘toenail cutters’ and therefore not the first point of contact for more complex cases that may require the prescription of scheduled medicines.

*“So, at the moment there's not many podiatrists that do it and I guess it's not something that the public is aware of... if you talk about podiatrists, you assume…general nail care. And most clients think you only do that... That's already like a stigma with podiatry. And the public not having knowledge that we can prescribe... We're not the first point of contact for anything related.”* ***P8*** *(non-endorsed; neutral; private sector; metro based)*

Being in rural locations offered many barriers including: access to mentors, time away from work, professional isolation, staffing, and internet access.

*“But as far as the logistics of studying these sort of things, we have a limit of where I live on a farm, we have limited internet access, so you know, online lectures and that sort of stuff would be a real burden for that process. So I travel around a lot, so there's a lot of time spent in cars and stuff, which take away time from them [family] as well as work. So yeah, there's lots of impediments to rural practice and my situation”* ***P7*** *(non-endorsed; unwilling to become endorsed; private sector; regional/rural based)*

#### Theme 3: extrinsic motivators

Time was a particularly notable barrier for private sector podiatrists in the rural setting as their workday involves a considerable amount of travel time, often in addition to their regular work hours. Similarly, on-endorsed podiatrists found the time required to complete training a barrier.

*“Well it's probably just not worth it from the amount of time involved for this. Maybe three to six months to treat would then maybe become more realistic to do.”* ***P10*** *(non-endorsed; neutral; private sector; metro based)*

Hospital based podiatrists who were currently undertaking training recognised the advantage they had of time not being a barrier for them.

*“…I was already doing this work [in hospitals] and already gaining hours and I didn't have to take time off of work to get hours…”* ***P2*** *(completed training; public sector; metro based)*

Personal priorities and stage of life and career also seem to play an influential role, as participants reported weighing up a number of personal life factors.

*“it depends on your stage of life as well. So, if you have commitments, family, children, a mortgage or so on so forth, that might also determine whether you go for it or not.”* ***P8*** *(non-endorsed; neutral; private sector; metro based).*

One participant reflected that as they were at the start of their career, they would like to settle in before undertaking further training. Conversely some commented that as they were at the end of their careers, they had already done other advanced study and had other interests they would rather invest their time in.

Participants were not only concerned about their own time, but that it would be more time efficient and convenient for the patient if they could prescribe directly rather than having to refer patients to their GP for an additional appointment.

*“…the convenience of it, that it would just make probably life a little bit easier for us and also for our patients. That sometimes it's quite annoying to them that they come and see me, and I say, ‘Oh, you need this and this, but I can't actually prescribe it to you. You need to now go and make an appointment and see your GP.’ And they just can't always get to see their GP on today or get in touch with their usual doctor.”* ***P3*** *(in-training; public sector; metro based).*

The barriers of time and stage of life were compounded by the financial costs involved in training, particularly for those who had to complete an approved podiatric therapeutics course.

*“There is also the financial…I believe it's about $5,000 to do that course… …..”* ***P6*** *(non-endorsed; willing to become endorsed; private sector; metro based)*

*“…because I've just moved house and getting a new mortgage and everything costs is a bit more prohibitive to me at the moment…”* ***P9*** *(non-endorsed; unwilling to become endorsed; private sector; regional/rural based)*

Interestingly, both the cost of training as well as the lack of financial incentives to become endorsed were reported as a disincentive by a podiatric surgeon who was endorsed, and therefore had considerable insight into the true financial and time commitment.

*“… there's a financial disadvantage…..if it's not going to make a massive difference to your podiatry practice… why spend the time and all the money to train yourself into something that's going to make very little difference to your day to day practice.”* ***P1*** *(endorsed podiatric surgeon; private sector; metro based)*

Participants repoted the lack of access to Pharmaceutical Benefits Scheme (PBS) funding for podiatrists meant their patients do not qualify for existing subsidies and must pay full price for medications prescribed by a podiatrist. Podiatrists are the only non-medical endorsed prescribers not covered by the PBS. The additional costs to patients were a barrier for some participants, as was the difficulty this created for funding hospital inpatients.

*“...the lack of PBS funding means it's difficult for podiatrists to prescribe, even if they're endorsed because the pharmacists are looking at them going, ‘Well, you don't have a prescriber number, so how can you prescribe?’ The pharmacy people in the hospital departments don't know where to get the funding for. Somebody's got to pay for the drugs. If you work in a hospital or somewhere else, where does the money come from? So, the lack of PBS is a major concern”* ***P1*** *(endorsed podiatric surgeon; private sector; metro based)*

Some participants thought being and endorsed prescriber could be recognised with higher private or public health rebates. However, the desire for autonomy outweighed the finance disincentives for some.

*“…so I could say that if you're an endorsed provider you get…better Medicare rebate where you can get rebates and things like that…”* ***P9*** *(non-endorsed; unwilling to become endorsed; private sector; regional/rural based)*

*“There’s no financial gain… The gain is in a more complete management portfolio…”* ***P1*** *(endorsed podiatric surgeon; private sector; metro based)*

Another interesting finding from this research was the lack of awareness of the current training requirements.

*“Probably my slight lack of understanding is probably also just causing me not to make a concrete plan to move forward.”* ***P6*** *(non-endorsed; willing to become endorsed; private sector; metro based)*

This was demonstrated by some participants who were unaware that recent changes to Pathway B for endorsement decreased the number of case studies required to 15.

*“… if it's a hundred cases then that's going to take a long period of time…I'd probably think it might not be worth becoming endorsed. If it was more, 10 to 12 cases, but then it might become more feasible to achieve that in a relatively short period of time. “****P10*** *(non-endorsed; neutral; private sector; metro based)*

Some participants also provided suggestions on changes as a means of ameliorating some of the barriers reported previously. One participant reflected that an increase in community acceptance, PBS funding, or numbers of endorsed prescribers would occur in the near future and may drive increased uptake of endorsement.

*“…I think that in the next five years there'll be exponential growth…it grows because it's got more cultural acceptance in the community, PBS funding, more people are out there practicing, [and] train people. I think that will change”* ***P1*** *(endorsed podiatric surgeon; private sector; metro based)*

Additionally, there was support for the approved qualification in podiatric therapeutics and all requirements for the endorsement qualification to become incorporated into the undergraduate course.

*“I think it's a positive thing and I think it should probably be in the Uni course as much as it can be before a person graduates ....”* ***P9*** *(non-endorsed; unwilling to become endorsed; private sector; regional/rural based)*

Further to this, one participant reflected that unexpected global health events, such as the SARS-CoV-2 (COVID-19) pandemic may facilitate his motivation to become an endorsed prescriber.

*“I think it's something like what's happening at the moment [COVID-19], like if things came along or conditions came along that needed to be prescribed drugs, then maybe that would convince me…”* ***P7*** *(non-endorsed; unwilling to become endorsed; private sector; regional/rural based)*

## Discussion

While nearly a decade has passed since Australian podiatrists and podiatric surgeons were able to use a range of drugs to treat podiatric conditions, the uptake for endorsed for scheduled medicines (endorsed prescribers)  among podiatrists remains low. To date, no research has explored the barriers to and facilitators for becoming endorsed, therefore this research aimed to address this knowledge gap. Findings from this research demonstrated that important motivators for podiatrists to become endorsed prescribers are the ability to offer complete patient care and improved patient outcomes. However, whether podiatrists became endorsed prescribers was influenced by social and workplace contexts. Working with those who hold endorsement was a critical facilitator demonstrating the importance of modelling behaviours and how this can result in individuals integrating and normalising professional behaviours into their own practice.

An important finding to emerge from this research was the limited access to mentors and supervised practice opportunities. This played a pivotal role for many participants and is reflective of the limited number of podiatrists that currently hold endorsed prescriber status. The considerable barrier of access to these opportunities is perhaps a symptom of a lack of formalised structure in the mentoring and supervised practice component of the current Pathway B to endorsement. Similar findings have been reported in other allied health literature, such as advanced and extended scope of physiotherapy [25] and allied health assistants [26], which have identified varied and diverse training opportunities for these discipline groups.

The lack of understanding of the current training requirements to become an endorsed prescriber demonstrated by some participants may be due to a range of reasons. First, while the role of an endorsed prescriber was established more than a decade ago, this may not have been matched with considerable and consistent awareness raising initiatives with the podiatry discipline. Second, given there are currently only a small number endorsed prescribers, there may not be sufficient critical mass of podiatrists to promote and advertise this role to the wider professional. Finally, the lack of understanding of what is required to become an endorsed prescriber may arise from a genuine lack of interest in this specialised role.

A group at particular disadvantage were rural podiatrists. Some rural podiatrists were highly motivated to become endorsed prescribers, where they could potentially play an important role in addressing difficulties faced by patients’ access to GPs. Lack of access to health care services in rural areas have been well documented [27], including allied health [17]. Yet, in addition to the barriers faced by urban podiatrists this group experienced barriers such as poor internet connection, lengthy travel times for both work and to access education opportunities, and a lack of staff to provide backfill for time lost. These findings are supported by previous literature which has explored allied health professionals perspective of engagement with professional development opportunities [19, 28]. Further compounding these issues are system-level barriers, such as historical funding arrangements, which means patients who are treated by endorsed podiatrists do not qualify for government subsidy (i.e., PBS) or Medicare Safety Nets [22]. Being the only non-medical prescribers not eligible to be PBS prescribers is a significant barrier for podiatrists. This means patients are likely to be out of pocket for their prescription costs, which may be a disincentive for them to seek consultation from an endorsed podiatrist and may even see the podiatrist refer to the GP for the prescription, negating one of the purposes for establishing non-medical prescribers.

From these findings several recommendations could be made to enhance podiatrists’ engagement with endorsed prescribing. The intent to enable podiatrists to complete their undergraduate training as endorsed prescribers will be a significant development. For those required to complete the endorsement training as it currently stands, the findings of this study suggest providing adequate mentors and workplace role models were strong facilitators. Apart from increased opportunities to meet the training requirements, this finding is consistent with SDT that increased social exposure assists in establishing prescribing as a key component of podiatry practice.

With the current interest in lifelong learning amongst Australian policymakers [29], the extensive experience pool among currently registered podiatrists should be acknowledged as a valuable resource. Investment in supporting a group which has demonstrated commitment to the profession to become endorsed prescribers will allow a more rapid adjustment to change. Building a large pool of podiatrists who are endorsed prescribers, in addition to providing mentors, will provide role models and social support that could motivate behaviour change and normalise the professional expectation of this skill. Achieving a ‘critical mass’ of endorsed prescribers could create a turning point in professional expectations that favours endorsement. Growth of this sector will also enable robust cost versus benefit analysis, potentially aiding in achieving PBS prescriber rights. Moreover, more visible endorsed prescribers may improve public awareness that podiatrists are able to prescribe scheduled medicines, which in turn could increase demand for this service.

Apart from investment in training, increased private health and Medicare rebates to endorsed prescribers could be considered to incentivise podiatrists, similar to those offered to general practitioners for mental health and vocational health training. Quite a few participants did not perceive adequate gain for the financial and time costs paid. Financial rewards may assist podiatrists who do not see adequate financial return on their investment in the endorsement training program. Additional payments to support rural podiatrists to upskill, for example along the lines of the special incentive payments offered to rural GPs such as the Workforce Incentives Program [30], could ameliorate some of the heavy burden of financial barriers that this group confronts. The findings of this research provide learning opportunities for other non-medical professions currently attempting to gain endorsement, such as physiotherapy. Physiotherapists have identified similar priorities (such as training and educational opportunities) for the successful implementation and uptake of endorsement [31]. The identification of a commonality of needs across professions attempting to gain endorsement suggests a collaborative approach may be of benefit. For example, this could occur in the form of interdisciplinary training opportunities using real-life case studies, replicating what might occur in future clinical practice settings.

As with any research, this study has limitations. Given that there were no established databases of podiatrists who have completed, currently undertaking or interested in endorsement training, the research team relied on personal and professional networks to “spread the word” and assist in recruitment. While the final sample does include podiatrists with different characteristics, the potential for missing key respondents needs to be acknowledged. Despite the research team’s best efforts, only three participants from rural and regional Australia participated in this research, while there are fewer podiatrists in rural and regional areas, given the potential impact of endorsed prescribers in these areas (as means of addressing persistent health care access issues), deeper exploration of these issues amongst a larger number of podiatrists from rural and regional areas would have been useful. Therefore, further research is recommended with this stakeholder group.

In conclusion, this study has contributed new understanding by identifying that workplace and social networks where behaviours could be modelled were effective facilitators to becoming an endorsed prescriber. Additionally, the negative impact of a lack of access to mentors and supervised training opportunities may be diminished by a more formal structure for the endorsement pathway. The findings highlight the advantage that hospital-based podiatrists have in opportunities to undertake supervised training. The authors suggest that investment in upskilling established podiatrists and financial incentives for endorsed prescribers may be the most rapid method of building a larger pool of endorsed podiatrists. Further research could explore the level of interest in undertaking training in this group, the most influential incentives to become an endorsed prescriber, and the unique circumstances of rural podiatrists.

## Data Availability

The datasets used and/or analysed during the current study are available from the corresponding author on reasonable request.

## Abbreviations

AHPRA: Australian Health Practitioner Regulation Agency
QD: Qualitative description
SDT: Self-determination theory
PBS: Pharmaceutical Benefits Scheme
GP: General practitioners
SA: South Australia
VIC: Victoria
QLD: Queensland
WA: Western Australia
